# Assessment of work-related Asthma cases: Our three-year experience

**DOI:** 10.12669/pjms.335.12923

**Published:** 2017

**Authors:** Ayse Coskun Beyan, Nur Safak Alici, Arif Cimrin

**Affiliations:** 1 Ayse Coskun Beyan, Dokuz Eylul University Faculty of Medicine, Occupational Medicine Department, Izmir, Turkey; 2 Nur Safak Alici, Dokuz Eylul University Faculty of Medicine, Occupational Medicine Department, Izmir, Turkey; 3 Arif Cimrin, Dokuz Eylul University Faculty of Medicine, Occupational Medicine Department, Izmir, Turkey

**Keywords:** Occupational asthma, PEF monitoring, Skin-prick test, Work exacerbated asthma, Work related asthma

## Abstract

**Objective:**

Work-related asthma (WRA) is one of the most common occupational diseases. In this study, we aimed to review diagnosing procedures and the characteristics of patients who were diagnosed with WRA.

**Methods:**

Between November 2013 and June 2016; 214 patients were referred to our clinic with WRA suspicion by an occupational health specialist, personal visit, chest disease specialists [61 (28%), 51 (23%), and 102 (47%) respectively]. Occupational history, functional and radiological assessment, skin prick test, PEF monitoring were done.

**Results:**

Fifty-four patients (25%) were diagnosed with OA, and 24 (11%) with WEA, total 78 workers were diagnosed with WRA. Twenty-five (32.1%) had allergic rhinitis, 13 (16.7%) had allergic dermatitis, and 8 (10%) had both diseases.

**Conclusion:**

WRA can be seen in many areas. Complaints are the basic route for admission to physician, and the diagnosis can be delayed for a long time as one year. Lower rates of referral by occupational health physicians are the signs of limitations on management of cases. Non-specific BPT and skin prick test for selected cases would be sufficient besides occupational history and clinical examination for the diagnosis of WRA. PEF assessment, one of the most important tests for the diagnosis of WRA, must be performed.

## INTRODUCTION

Occupational asthma which accounts for 9 to 25% of the adult asthma cases is one of the most common occupational diseases (OD).^1^,^2^ More than 360 substances are known to be related with work-related asthma WRA.^3^ Recently, (WRA) is classified into two groups as occupational asthma (OA) and work-exacerbated asthma (WEA). The first is divided into two groups as immunological asthma characterized by a latent time period after a sensitizer substance exposure and irritant-induced asthma characterized by a latent period after a high-dose irritant substance exposure. Work-exacerbated asthma is worsening of asthma after the irritant exposure at working place.^4^,^5^

There is no reliable statistical data for WRA in Turkey. According to the annual statistical data for 2014 of Social Security Institution (SSI), (OA/total OD: 6/494) six cases were accepted as OA.^6^ However, in limited number of studies conducted for WRA in Turkey, Elci et al.^7^ reported 14.6% among hairdressers, Turgut et al.^8^ reported 3.5% among auto and furniture painters, Temel et al.^9^ reported 22% among welders and 18% among dyers, and Kose et al.^10^ reported WRA as 4.2% among healthcare professionals.

In this study, we aimed to review the characteristics of patients who were diagnosed with WRA at Dokuz Eylul University (DEU), Department of Occupational Health (DOH) one of the authorized units for official OD.

## METHODS

The study protocol was approved by DEU Ethics Committee. Most of the cases which were admitted to our clinic were from Izmir, Manisa, Usak provinces. In these cities, total number of workers is 178.024 from the industrial branches that have potential asthma risk such as mining and metal business, textile product manufacture, leather and soon.^6^ All patients admitted to DEU DOH between November 2013 and June 2016, and diagnosed with OA and WRA were included. No sample selection was carried out.

### Diagnosis algorithm

According to the Global Initiative for Asthma (GINA) guideline, patients with defined asthma criteria were diagnosed with WRE per algorithm.^11^-^13^

### Functional assessment

Functional assessment was performed on the day after working day between 09:00-11:00 A.M. Spirometric measurement, reversibility test and BPT Sensor Medics V max. 22 0.6-2b versions was performed with spirometry device according to ATS criteria.^14^

### BPT

ATS protocol was used for the test.^15^ Non-specific BPT for resting period was performed during at least 10 days preferably 14 days after the period away from work.^16^

### Test utilization

Cases with working day(+), resting(-) or three-fold or more difference for PC20 dose between working day or resting was interpreted as compatible test result for OA. If both working day and resting were (+) test, result was interpreted as compatible with WEA. If working day (-), resting (+) and technically unacceptable tests were evaluated as incompatible for WEA diagnosis.^16^

### Skin prick test

Turkish Dermatology Association protocol was used for test and the test was assessed by dermatologist.^17^ Occupational history was gained with questioning the job(s) starting from the first job, used material(s), duration, time, place properties chronologically and extensively.^12^

### Assessment of PEF observation results

A graphic picture was created with the highest measurement of three measurements for each session on Microsoft Office Excel program. Daily PEF changes were calculated separately working and resting days. For daily changes [(PEFR max - PEFR min)/(PEFR max + PEFR min) x 0.5] x 100 was used. At least one 20% and more changes were accepted as significant.^18^ The PEF assessment graphic and job history were interpreted.^19^

During PEF:

a1. No significant daily changes during resting days and 20% and more changes during working days or

a2. Significant and continuous reduce in PEF values during resting period comparing to the working period was classified as ***‘Compatible with OA’*.**^14^

b. While daily regular changes during working days were present, presence lower but continuous changes for resting days comparing with working days was classified as ***‘Compatible with WEA’*.**^14^

c. Recording mistakes by patients, recording unsuitable results or presence of misfeasance was classified as ***‘technically unapproved(unsuitable)’***

### Diagnostic measurements

### Occupational Asthma

*Immunological Asthma:* Classification was performed as the complaints initiated after a time period of starting to work for the cases that had no complaint before; complaints exacerbated with work; a1 and a2 properties that were detected during PEF observation.^14^

### Irritant-related asthma

Onset of respiratory complaints after exposure of lower density irritant substance for a long time period or one intensive exposure for the cases that had not any complaint before; patients were classified as a1 and/or a2 properties that were detected during PEF observation.^14^

### Compatible with WEA: Presence of

“b” property during PEF observation of the patients with asthma diagnosis before, presence of sensitizer history that increases respiratory complaints outside the working place.^14^ Statistical analysis was performed using SPSS version 18.0 software (SPSS Inc., Chicago, IL, USA). Descriptive data were expressed in mean, median, minimum, maximum, and standard deviations.

## RESULTS

A total of 214 patients were admitted to our clinic with WRA suspicion. They were referred by an occupational health specialist, SSI (by personal visit), second- or third level chest disease specialists [61 (28%), 51 (23%), and 102 (47%) respectively]. Demographic data of a total of 78patients who were diagnosed with WRA are shown in Table-I.

**Table-I:** Sociodemographic data and referral information’s of cases.

		*n: 78(%)*
Age (Mean ± SD, min - max)		37.5±6.3 (21-52)
Sex	Male	54 (69.2)
	Female	24 (30.8)
Marital status	Married or having partner	68 (87.2)
	Single or divorced	10 (12.8)
Educational status	Secondary education or less	58 (74.4)
	High school and more	20 (25.6)
Smoking	Yes	29 (37.2)
	No	28 (35.9)
	Quitted	21 (26.9)
Amount of cigarettes (pack/year) Mean ±SD (min - max)		11.8±16.8 (4-120)
Chronic disease	Yes	6 (7.7)
	No	72 (92.3)

Most of the patients had at least one complaint of difficulty of breathing, wheezing, cough, rustling noise at the time of admission. The mean duration of complaints was 54.1 month (range, 1 to 200 months). Complaints of 74 (94.9%) cases were exacerbated at work place. A total of 75.6% of cases had at least one of the findings of expiratory rhonchus with physical examination on admission, and prolonged expirium. Twenty-five (32.1%) had allergic rhinitis, 13(16.7%) had allergic dermatitis, and 8(10%) had both allergic rhinitis and dermatitis. One of these cases was from metal; three of them were from health, one from textile, one from cleaning sector. According to the spirometric measurements, the mean FEV_1_was 79.9% of expected value, FVC was 85% of expected value, and the ratio was 78.7%. Clinical and functional assessment results are present in Table-II.

**Table-II:** Clinical and functional assessment results.

		*n: 78 (%)*
Presence of complaint at the time of admission*	Yes	76 (97.4)
	No	2 (2.6)
Total duration of complaints (month) Mean ±SD (min - max)		54.1+49.3 (1-200)
The relation of complaints with job	Yes	75 (96.1)
	No	3 (3.8)
The time for the initial complaint **		
Mean ±SD (min - max)		47.2+45.1 (1-168)
Physical examination findings during admission ***	Yes	59 (75.6)
	No	19 (24.4)
Functional assessment results (Mean ± SD) (expected %) (min- max)		
FEV 1		79.9+16.2 (32-136)
FVC		85+13,8 (44-127)
FEV1/FVC		78.7+8.5 (57-93)
PEF		76.2+18.6 (37-125)

* At least one positive complaints of difficulty in breathing, wheezing, cough, rustling voice

** Work branches were classified as metal, chemistry, health, cleaning and others, the differences between groups were noted. One-way ANOVA, p:0,76

*** Expiratory rhonchus, decrease in breath sounds, prolonged expiration, at least one positive physical examination finding

A total of 68 of the cases (87.2%) were working on admission. The most common sectors were from cleaning with 13 (16.6%) and painting industry with 12 (15.3%). The cases between (1-9) were from textile, dental technician, ceramic, salt production, leather tanning, plastic injection, nursery, pumper and costumer representative sectors. The mean working duration was 34.6 months (range, 1 to 156 months). The results are shown in Table-III.

**Table-III:** Findings related work life.

		*n: 78*	%
Working status at the time of admission	Yes	68	87.2
	No	10	12.8
Sector of work	Industry	17	21.8
	Service	61	78.2
Total duration of work (month) Mean ±SD (min - max)		34.6±51 (1-156)	

The PEF assessment was not performed in 18 (23%) patients, as they had some difficulties at working place. The PEF examinations of 36 cases (46%) were not compatible with OA, 12 (15%) of them were not compatible with WEA, 12(15%) of them were not compatible with asthma. This assessment was useful for the diagnosis in 48 patients (61%).

In addition, BPT was performed for 75 (96%) cases. 16 (20%) cases had (+) BPT test for both working and resting; however, there was a significant difference for PC20 doses. Twenty-two (28%) cases had (-) BPT test during working period. BPT test was useful for the diagnosis of WRA in 37 (47%) cases. Specific BPT test was not performed for any case.

### OA diagnosis

The PEF examination + BPT were used for 30 (55%) of 54 (69%) cases diagnosed with OA. Despite PEF, observation is not compatible or technically not performed for 18 (20%) cases, OA diagnosed using BPT in combination with other diagnostic tools, and improvement of clinical condition after exposure discontinued.

### WEA diagnosis

Twelve (15%) of 24 patients (30.7%) diagnosed with WEA were diagnosed with WEA by PEF assessment. Work-exacerbated asthma was diagnosed based on the observation of the relation of complaints with work in 12 (15%) cases. Status reports for cases were developed and sent to control unit. The results are shown in Table-IV.

**Table-IV:** Findings related with diagnosis period.

	*N: 78 (%)*
*Comments for PEF observation*	
Compatible with OA	36 (46.2)
Compatible with WEA	12 (15.4)
Unreliable data	12 (15.4)
Not observed	18 (23.1)
*BPT*	
Performed used for WRA diagnosis	53 (67.9)
Performed not used for WRA diagnosis	22 (28.2)
Not performed	3 (3.8)
*Other tests*	
Prick test positivity	6 (12.8)
Presence of allergic dermatitis	15 (31.9)
Presence of allergic dermatitis	9 (19.1)
*Latest diagnosis*	
OA	54 (69.2)
WEA	24 (30.7)

## DISCUSSION

A total of 36% of 214 patients who were referred with the suspicion of OA were diagnosed with WRA. Of these, 69% were OA, and 30% were WEA, indicating that unlike the official statistics in Turkey. WRA is still seen in risky branches.^6^ According to the literature, higher WRA detection rates in our study can be explained by the fact that our clinic is a tertiary reference clinic.^1^,^2^,^10^ Only the patients referred by another physician or with SSI referral are accepted by our clinic. High diagnosis rates of OA would be due to the examination of the admitted cases by at least one physician who is familiar with occupation and health relation. On the other hand, nearly half of the WRA diagnosed patients referred by chest diseases specialists and occupational health specialists referred one third of them. Despite expectation for referral of more patients would by occupational health specialist that are in the first line of occupational health service, less patients were referred comparing with chest disease specialists and this condition was taught as a reflection of limitations.

The mean age at the time of diagnosis was 37.5 years in our study. As similar to a limited number of studies conducted in Turkey on different industrial branches, cases were consisting of young individuals and those with basic education.^6^,^7^

At the time of admission, nearly all the cases had respiratory complaints and physical examination findings that were supporting asthma. This result suggests that respiratory complaints are the main causes. The mean duration before the onset of complaints was 47 months, while the mean admission period for diagnosis was 54 months. Serious functional loss was not seen with spirometric measurements during the admission of patients. This condition can be explained with ignorance of complaints by patients and chronicity of this problem and not applying for medical support. Another important reason is that prolonged application period would be limited or problematical communication with occupational health specialists.

The patients who were admitted were from cleaning and painting sectors. According to European Community Respiratory Health Survey, 15,637 cases were assessed and it was stated that the first four sectors that cause WRA were farmers, painters, plastic and cleaning sectors. Results are compatible with literature.^20^ The cases were from different industrial branches as table salt production, leather tanning, plastic injection and customer representatives in other studies. These sectors are less known in terms of OA. Customer representation would be classified in building-related disease group.^21^ However the most important limitation was that we had not performed sampling in working place so that would not be able show the cause(s) of asthma. Our study reveals new work branches that are risky for OA.

Furthermore, PEF assessment and non-specific BPT were sufficient for showing the relation with occupation after finalizing of asthma diagnosis as it was stated in WRA algorithm of AACP and as specific BPT was the reference standard for OA diagnosis.^11^,^22^

In our study, most useful test for diagnosis was PEF observation. There were problems for performing tests because of concerns for job security. Eighteen (23%) workers rejected observation for this reason. The second most useful test was non-specific BPT.

Immunological tests were useful for diagnosis as stated in EAACI report. Ig E and skin prick test were used for the cases with high-molecular weight substances exposure like latex.^23^

### Limitations of the study

In the diagnosis of asthma, responsible factor was defined with only declaration. Due to the official limitations, occupational hygiene measurements could not be performed. Other information related with working place and presence of air conditioning, usage of personal protective equipment, product substance security information forms, previous health records were not collected. For this reason, WRA subgroup distinction was performed on the basis of history. Specific bronchial provocation tests would not be performed for the patients that PEF observation was not performed or would not be performed because the patient had left the job. A bias related with selection would be seen and these results cannot be generalized because this information was only gained from the patients that were admitted to our outpatient clinic. Therefore, the cases diagnosed in our clinic were reported to the Ministry of Health and the Ministry of Labor and Social Security, and occupational health physicians were warned about reducing and preventing exposure, re-defining the risk areas for WRA.

## CONCLUSION

The present study shows that WRA can be seen in many sectors. Complaints are the basic route for admission to physician, and the diagnosis can be delayed for a long time period up to one year. Lower rates of referral by occupational health physicians are the signs of limitations on the observation of health and defining and management of cases. Awareness of occupational health physicians must be raised by paying attention to that delay, health observation in working places and improvement of case managements are necessary. Priority education programs must be planned for occupational health physicians especially about the risky industrial branches as painting, cleaning, dental technicians and health care professionals. PEF assessment, one of the most important tests for the diagnosis of WRA, must be taught to occupational health physicians, observations must be performed by occupational health physicians PEF observation, non-specific BPT, and skin prick test for suitable cases would be sufficient besides occupational history and clinical properties for the diagnosis of WRA.

### Authors` Contribution

**ACB, NSA, AHC** conceived, designed and did statistical analysis & editing of manuscript.

**ACB** did data collection and manuscript writing.

**ACB** takes the responsibility and is accountable for all aspects of the work in ensuring that questions related to the accuracy or integrity of any part of the work appropriately investigated and resolved.
